# A Text-Book of Surgical Principles and Surgical Diseases of the Face, Mouth, and Jaws, for Dental Students

**Published:** 1903-09

**Authors:** 


**A Text-book of Surgical Principles and Surgical Diseases of the Face, Mouth, and Jaws, for Dental Students.** By H. HORACE GRANT, M.D. Pp. 231. London: W. B. Saunders & Company. 1902.

A book which gives to dental students an outline of those pathological processes, and general surgical diseases, which every dentist should know something about, must be a useful one, and this work does clearly, though very briefly, describe them. But why should a dental surgeon be taught the nature and treatment of diseases and injuries in the region of the face, mouth, and jaw because he treats those of the teeth? Why should deformities of the nasal system, and lymphadenoma of the cervical glands be considered suitable for the dental surgeon's attention rather than a bowed tibia or a tumour of the testis? If all such subjects had been omitted and fuller details of the nature of inflammation and suppuration, and other pathological processes had been given, and the nature and complications of wounds more fully described, we think the work would be of more benefit to the dental student. We are glad to see a chapter on anæsthetics and hemorrhage, but why a method which could not be used for dental operations—spinal cocaine anæsthesia—should be described, or why nasal hemorrhage should be considered at all, we fail to see. If the examining boards in America require such knowledge of candidates the book may fulfil a most useful purpose, but we venture to think that if so, the examining boards require the student to waste his time over the study of what he will never have sufficient knowledge to turn to good account. If the dental surgeon is to be a face, mouth, and jaw surgeon, or even a mouth and jaw surgeon alone, let his title be changed, and the public will understand the kind of specialist they may consult.

